# Src kinase inhibition restores E-cadherin expression in dasatinib-sensitive pancreatic cancer cells

**DOI:** 10.18632/oncotarget.26621

**Published:** 2019-02-01

**Authors:** Austin R. Dosch, Xizi Dai, Alexander A. Gaidarski III, Chanjuan Shi, Jason A. Castellanos, Michael N. VanSaun, Nipun B. Merchant, Nagaraj S. Nagathihalli

**Affiliations:** ^1^ Division of Surgical Oncology, Department of Surgery, University of Miami Miller School of Medicine, Miami, FL, USA; ^2^ Sylvester Comprehensive Cancer Center, University of Miami, Miami, FL, USA; ^3^ Department of Pathology, Vanderbilt University School of Medicine, Nashville, TN, USA; ^4^ Department of Surgery, Vanderbilt University School of Medicine, Nashville, TN, USA

**Keywords:** pancreatic ductal adenocarcinoma, SRC kinase, E-cadherin, Dasatinib, epithelial-to-mesenchymal transition (EMT)

## Abstract

The Src family of non-receptor tyrosine kinases are frequently activated in pancreatic ductal adenocarcinoma (PDAC), contributing to disease progression through downregulation of E-cadherin and induction of epithelial-to-mesenchymal transition (EMT). The purpose of this study was to examine the efficacy of Src kinase inhibition in restoring E-cadherin levels in PDAC. Immunohistochemical analysis of human PDAC samples showed Src activation is inversely correlated with E-cadherin levels. Protein and mRNA levels of E-cadherin, the gene expression of its various transcriptional repressors (Zeb1, Snail, Slug, LEF-1, TWIST), and changes in sub-cellular localization of E-cadherin/β-catenin in PDAC cells were characterized in response to treatment with the Src inhibitor, dasatinib (DST). DST repressed Slug mRNA expression, promoted E-cadherin transcription, and increased total and membranous E-cadherin/β-catenin levels in drug-sensitive PDAC cells (BxPC3 and SW1990), however no change was observed in drug-resistant PANC1 cells. BxPC3, PANC1, and MiaPaCa-2 flank tumor xenografts were treated with DST to examine changes in E-cadherin levels *in vivo*. Although DST inhibited Src phosphorylation in all xenograft models, E-cadherin levels were only restored in BxPC3 xenograft tumors. These results suggest that Src kinase inhibition reverses EMT in drug-sensitive PDAC cells through Slug-mediated repression of E-cadherin and identifies E-cadherin as potential biomarker for determining response to DST treatment.

## INTRODUCTION

Pancreatic ductal adenocarcinoma (PDAC) is an aggressive malignancy with a 5-year survival rate of 8% [1]. This dismal prognosis is largely due to the propensity for PDAC to metastasize early within the disease course, as over half of all PDAC patients will have widespread metastases at time of diagnosis [2]. The ability for PDAC cells to metastasize is dependent on their ability to adopt a pro-metastatic phenotype. In a process known as epithelial-to-mesenchymal transition (EMT), cancer cells lose apical-basal polarity, downregulate cell-cell adhesion pathways, and adopt characteristics of mesenchymal cells which promotes cellular motility and enhances their invasive properties [3]. A hallmark of EMT is the loss of E-cadherin, a major cell-cell adhesion protein and marker of epithelial differentiation in PDAC. Previous studies have reported downregulation of E-cadherin expression is controlled by numerous transcription factors which promote EMT, including Snail, Slug, Zeb1, TWIST, and LEF-1 [4–7]. These transcription factors are downstream products of several kinase pathways which are constitutively active in PDAC. In an attempt to target multiple pathways simultaneously, recent studies have turned attention to the proto-oncogene c-Src (Src). Src is a non-receptor tyrosine kinase which regulates the action of multiple G protein-coupled receptors, receptor tyrosine kinases, and focal adhesion kinase (FAK), which promote activation of numerous divergent pathways [8–10]. The Src kinase family is overactive in colon, liver, lung, breast, and PDAC tumors and has been implicated in the activation of pro-survival pathways, stimulating angiogenesis, and promoting invasion [8]. Additionally, Src enhances the activity of multiple oncogenic signaling pathways including phosphatidylinositol 3-kinase/AKT, c-Myc, and the *Ras/*Raf pathway which are over activated in many PDAC cases [11, 12].

Through its diverse cellular functions, the Src kinase family has been recently identified as a potential mediator of cancer-associated EMT [13]. Src activation triggers the phosphorylation of β-catenin, a scaffold protein which complexes with the cadherin family of proteins. β-catenin is an essential structural compound which stabilizes cell-cell adhesion through the formation and maintenance of adherens junctions. Upon phosphorylation by Src kinase, β-catenin dissociates from membrane-associated cadherins and translocates to the nucleus, where it triggers the activation of multiple pro-EMT transcription factors that negatively regulate E-cadherin expression. These changes result in the disruption of cadherin-mediated cell-cell adhesion, thereby augmenting the migratory capacity of tumor cells [14, 15]. Loss of E-cadherin is the sentinel event in EMT and a strong contributor to the development of PDAC metastases [16]. However, treatment options to restore E-cadherin expression and suppress the EMT phenotype in PDAC are lacking.

Due to the frequency of Src dysregulation in PDAC, treatments aimed at disrupting this kinase cascade represents a promising therapeutic target for blocking EMT [17]. Dasatinib (DST), a potent FDA-approved oral inhibitor of Src family kinases, has been trialed in several studies in patients with solid organ malignancies [18–20]. However, no prior investigation to date has examined the potential benefit of DST in restoring E-cadherin expression and reversing EMT in PDAC and the mechanisms governing this process remain largely unknown. In this study, we have demonstrated that there is an inverse relationship between Src activation and E-cadherin expression in human patients, suggesting elevated Src activity is linked to E-cadherin suppression. Utilizing previously identified DST-sensitive and drug-resistant PDAC cell lines, we established the therapeutic efficacy of Src kinase inhibition in restoring E-cadherin and membranous β-catenin expression through suppression of the pro-EMT transcription factor Slug in drug-sensitive cells. Lastly, we demonstrated that DST treatment promotes epithelial differentiation by increasing E-cadherin expression in an *in vivo* xenograft model of PDAC. These changes were only seen in DST-sensitive cell lines, as the EMT phenotype in resistant cell lines was not affected by Src kinase inhibition *in vitro* or *in vivo*.

## RESULTS

### Activation of Src is inversely correlated with E-cadherin levels in human PDAC samples

**Figure 1:**
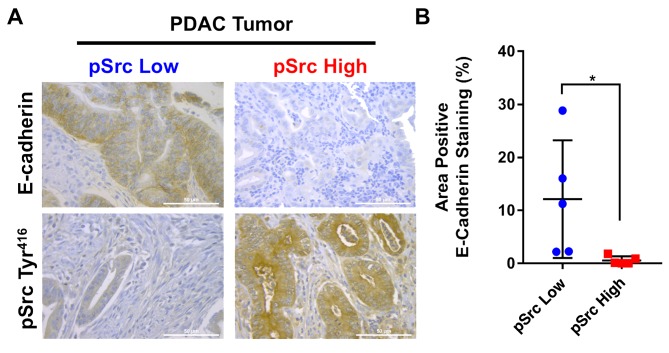
Inverse correlation between Src kinase activation and E-cadherin levels in human PDAC samples To determine if there is an association between elevated Src kinase activity and decreased E-cadherin expression, levels of E-cadherin in pSrc^low^ (N=5) and pSrc^high^ (N=5) human PDAC tissue specimens were compared by immunohistochemistry **(A)**. Images were analyzed as a percentage positive E-cadherin staining of total area in pSrc^low^ and pSrc^high^ patient tissues (scale bar = 50μm) **(B)**. ^*^p<0.05.

Activation of Src by phosphorylation of the Tyr^416^ residue triggers its biologic activity and leads to activation of numerous oncogenic pathways in PDAC which regulate E-cadherin expression [17, 21]. Analysis of a tissue microarray of human PDAC samples (N=50) with matched normal control pancreatic tissue (N=20) revealed that E-cadherin levels were markedly reduced in PDAC tumor specimens (Supplementary Figure 1A). To examine the association between activated pSrc levels and reduced E-cadherin expression, human PDAC tumor samples were analyzed by immunohistochemistry for pSrc and stratified into pSrc^low^ (N=5) and pSrc^high^ (N=5) groups based on tissue levels (Supplementary Figure 1B). In pSrc^low^ specimens, E-cadherin expression was markedly increased compared with levels in pSrc^high^ PDAC tumor samples (Figure 1A&1B). The results demonstrate that Src kinase activation is inversely correlated with E-cadherin expression in human PDAC samples.

### Src kinase inhibition restores E-cadherin and β-catenin levels and decreases cellular invasion in drug-sensitive PDAC cells

**Figure 2:**
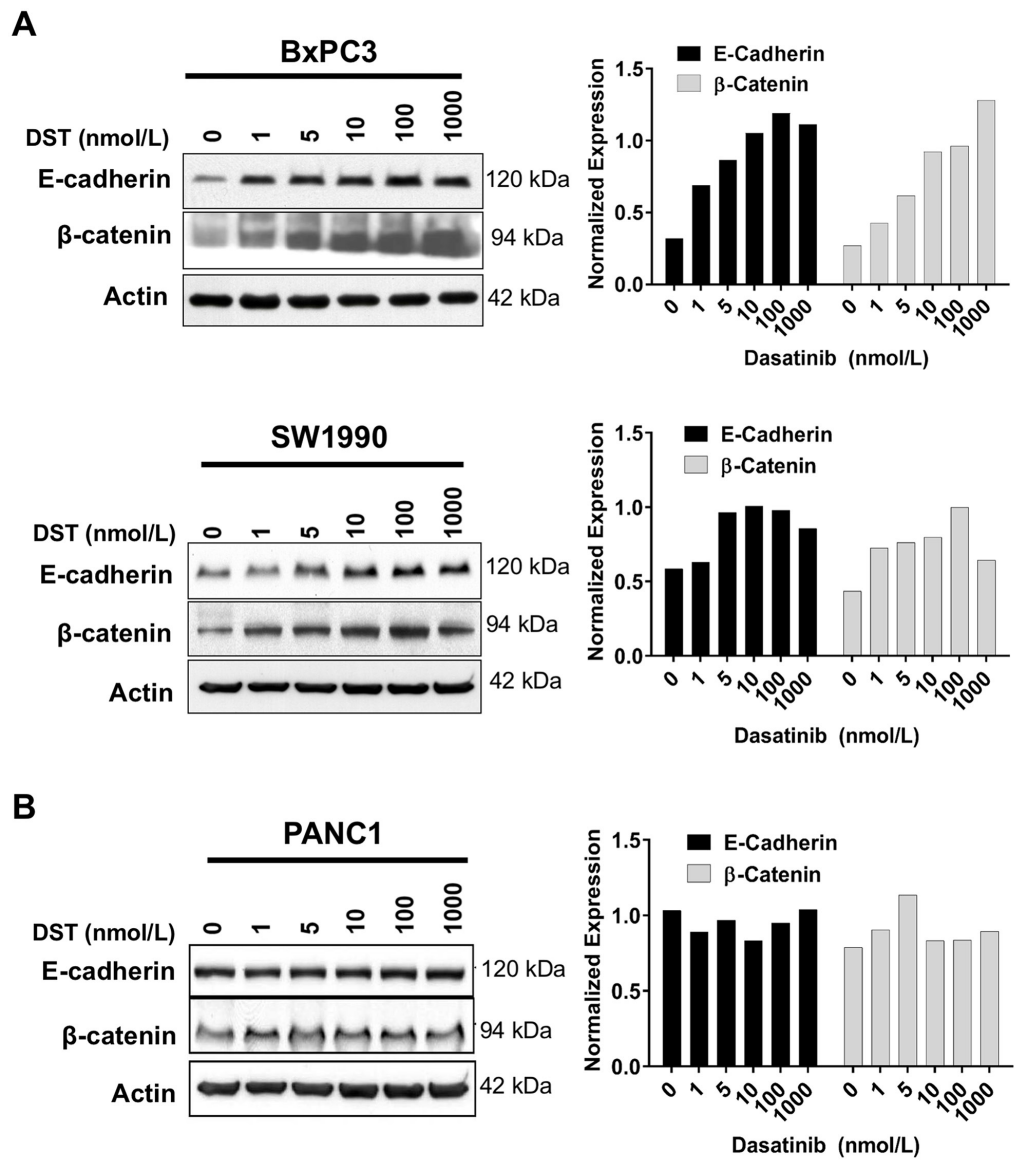
Src kinase inhibition increases E-cadherin and β-catenin protein levels in drug-sensitive PDAC cells Drug-sensitive **(A)** and resistant **(B)** PDAC cells were treated with DMSO or DST (0-1000 nmol/L) for 12 h, lysed, and analyzed by Western blotting with indicated antibodies. Western blots were quantified by densitometric analysis. Values are normalized to β-actin loading control.

After establishing that Src activation is inversely correlated with E-cadherin expression in human PDAC samples, we aimed to characterize the role of Src inhibition in restoring E-cadherin expression and reversing EMT *in vitro*. Previously, we have identified PDAC cell lines which are either sensitive (BxPC3, SW1990) or resistant (PANC1) to growth inhibition by DST [17]. Using these cell lines, E-cadherin and β-catenin expression in response to DST was evaluated by Western blot analysis. DST was effective in restoring E-cadherin and β-catenin protein levels in a dose-dependent manner in sensitive (Figure 2A) but not resistant (Figure 2B) PDAC cell lines.

**Figure 3:**
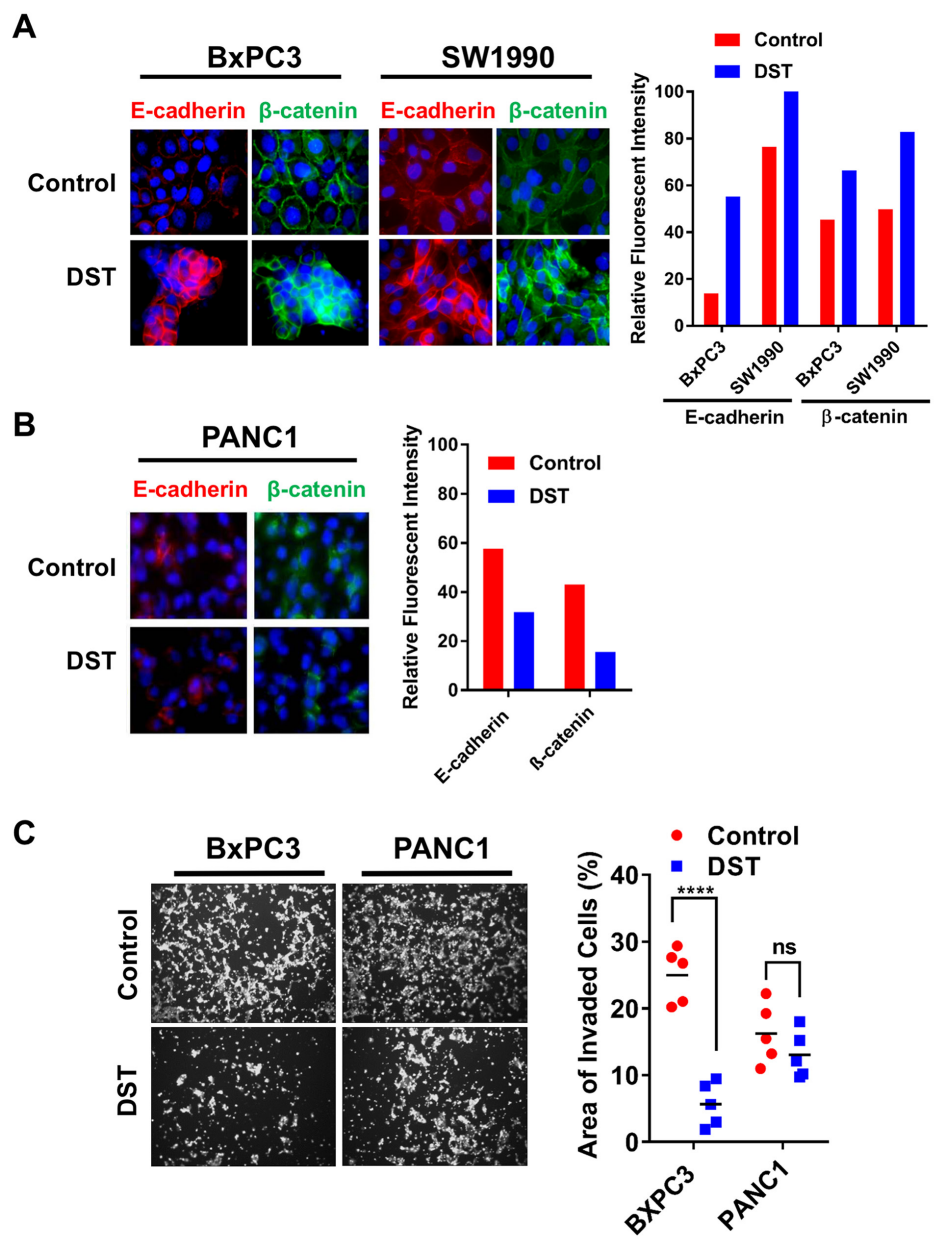
Src kinase inhibition restores membranous expression of E-cadherin and β-catenin and decreases cellular invasion in drug-sensitive PDAC cells Drug-sensitive **(A)** and resistant **(B)** PDAC cell lines were treated with DMSO or DST (10 nmol/L) for 12 h and analyzed by immunofluorescence with indicated antibodies. Fluorescent intensity of E-cadherin and β-catenin were quantified for each cell line in response to DST treatment. **(C)** To determine the effects of DST on inhibiting pro-metastatic properties in PDAC cells, drug-sensitive BxPC3 and resistant PANC1 cells were treated with DST (100 nmol/L) or equal volume of DMSO (control), subjected to matrigel invasion assay and quantified as area of invaded cells after 24 hours. ^****^p<0.0001, ^ns^ not significant.

To further examine the efficacy of Src kinase inhibition on reversing EMT, we treated both sensitive and resistant PDAC cell lines with DST and analyzed the presence and location of E-cadherin and β-catenin with treatment. Using immunofluorescent staining, we confirmed that total E-cadherin and β-catenin levels increase in response to DST treatment in sensitive cell lines (Figure 3A) without any appreciable change in the resistant PANC1 cell line (Figure 3B). Furthermore, Src kinase inhibition increased membranous localization of both E-cadherin and β-catenin in sensitive PDAC cells, a change which again was not seen in PANC1 cells. In accordance with these findings, DST treatment was effective in reducing invasion in BxPC3 but not PANC1 cells (Figure 3C), further highlighting the role of Src kinase inhibition in reversing the pro-metastatic properties of drug-sensitive PDAC cells.

### The pro-EMT transcription factor Slug is involved in Src kinase-dependent downregulation of E-cadherin expression in DST-sensitive PDAC cell lines

To investigate the mechanism of Src kinase inhibition on transcriptional upregulation of E-cadherin, we initially screened transcription levels of EMT-related genes (E-cadherin, LEF-1, Slug, Zeb-1, TWIST, and Snail) in response to DST treatment in both sensitive and resistant PDAC cells by qPCR. In BxPC3 cells, DST treatment resulted in suppression of the pro-EMT gene Slug, a negative transcriptional regulator of E-cadherin, along with a simultaneous increase in E-cadherin gene levels (Supplementary Figure 2A). There was no change in mRNA levels of other pro-EMT genes (LEF-1, Snail, Zeb-1, TWIST) produced by Src kinase inhibition in BxPC3 cells, suggesting that Slug is the putative transcription factor targeted by DST in drug-sensitive PDAC cells. Treatment with DST in drug-resistant PANC1 cells did not enhance E-cadherin transcription or reduce levels of LEF-1, Slug, Zeb-1, TWIST, or Snail (Supplementary Figure 2B).

**Figure 4:**
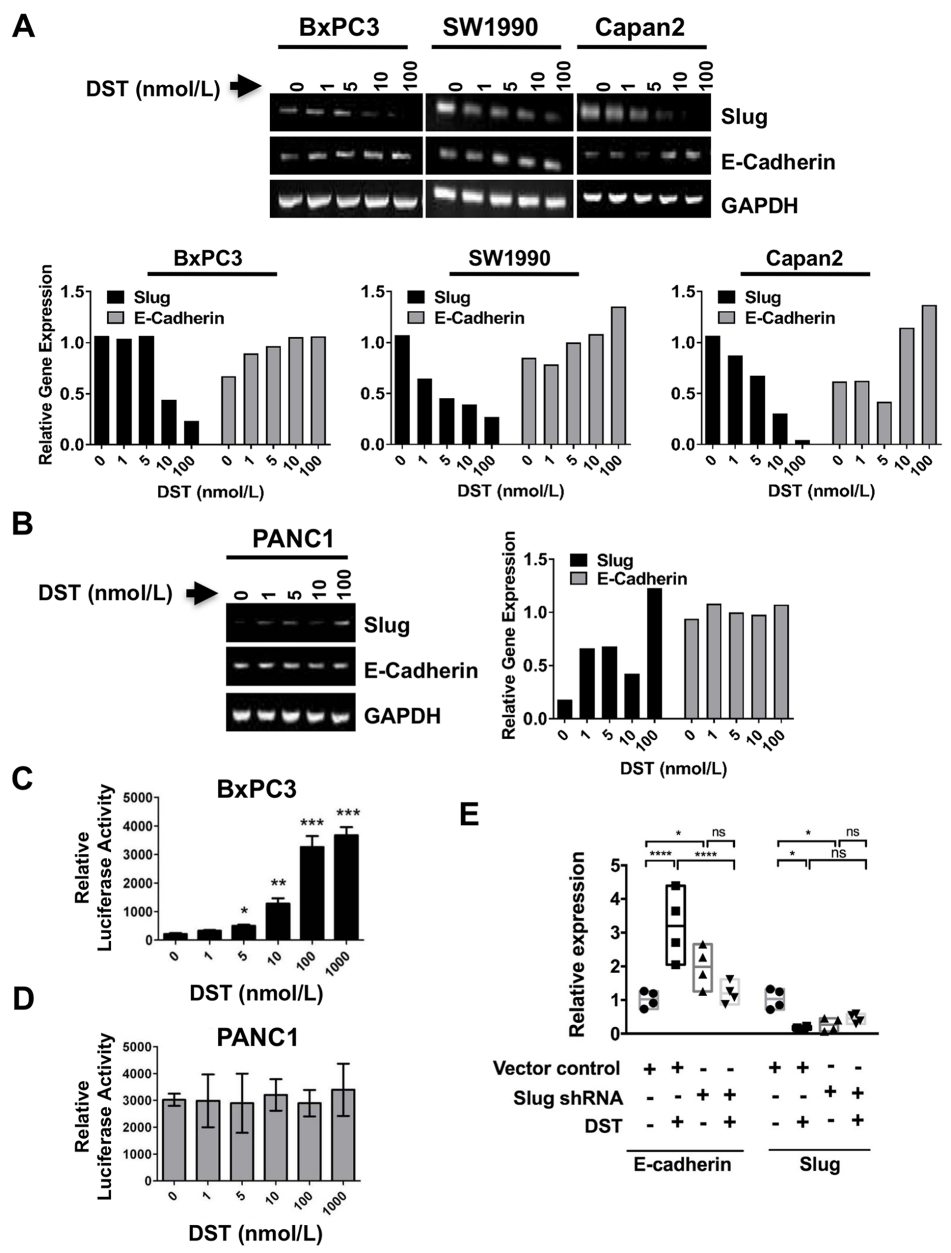
Src kinase regulates E-cadherin expression through the transcription factor Slug To verify whether targeting Src kinase can affect E-cadherin and Slug expression at mRNA level, RT-PCR was performed. PDAC cells were treated with DST in a dose-dependent manner (0-1000 nmol/L) for 12 h. RT-PCR analyses were performed to verify whether E-cadherin and Slug regulation by Src kinase inhibition occurs at the transcriptional level in drug-sensitive **(A)** and resistant **(B)** PDAC cells. Relative gene expression was calculated by normalizing each treatment value to corresponding GAPDH signal intensity, then reported relative to control signal intensity. Luciferase reporter assay for E-cadherin promoter activity in response to DST treatment (0-1000 nmol/L) in drug-sensitive **(C)** and resistant **(D)** PDAC cell lines was performed to determine if inhibition of Src kinase acts to enhance E-cadherin transcription in drug-sensitive cells. **(E)** To confirm Slug is essential for DST-mediated E-cadherin expression in drug-sensitive PDAC cells, BxPC3 cells were transfected with short-hairpin RNA (shRNA) to Slug and treated with DST (100 nmol/L). qPCR was performed to analyze relative expression levels of E-cadherin and Slug in response to shRNA knockdown and/or DST treatment. ^*^p<0.05, ^**^p<0.01, ^***^p<0.001, ^****^p<0.0001, ^ns^ not significant.

To validate that Slug expression is affected by DST inhibition in drug-sensitive PDAC cells, we utilized RT-PCR using total cellular RNA from both sensitive and resistant PDAC cells (Figure 4). E-cadherin mRNA expression was increased by DST treatment in a dose-dependent manner in sensitive PDAC cells (Figure 4A), whereas no change was observed in resistant PANC1 cells (Figure 4B). To further determine whether E-cadherin promoter activation is responsive to Src inhibition with DST, E-cadherin promoter activity was measured by conducting transient transfection assays in both drug-sensitive BxPC3 (Figure 4C) and resistant PANC1 (Figure 4D) cells. Cells were pre-treated with DST (0-1000 nmol/L) for 12 hours prior to transfection. E-cadherin promoter activity was increased significantly with DST treatment in a dose-dependent manner in drug-sensitive BxPC3 cells (Figure 4C), whereas no change was observed in the E-cadherin promoter activity for the resistant PANC1 cells (Figure 4D). These results confirm that inhibition of Src kinase increases E-cadherin expression at the transcriptional level in drug-sensitive PDAC cells.

To further delineate the role of Slug in regulating E-cadherin levels in drug-sensitive PDAC tumor cells, shRNA-mediated knockdown of Slug was performed in BxPC3 cells. Slug-knockdown in BxPC3 cells showed a significant increase in E-cadherin mRNA transcription, similar to the levels observed with pharmacologic Src kinase inhibition with DST (Figure 4E). These results implicate DST-mediated repression of Slug as a key mechanism in restoring E-cadherin levels in drug-sensitive PDAC cells.

### DST treatment restores E-cadherin expression in drug-sensitive PDAC cell lines *in vivo*

**Figure 5:**
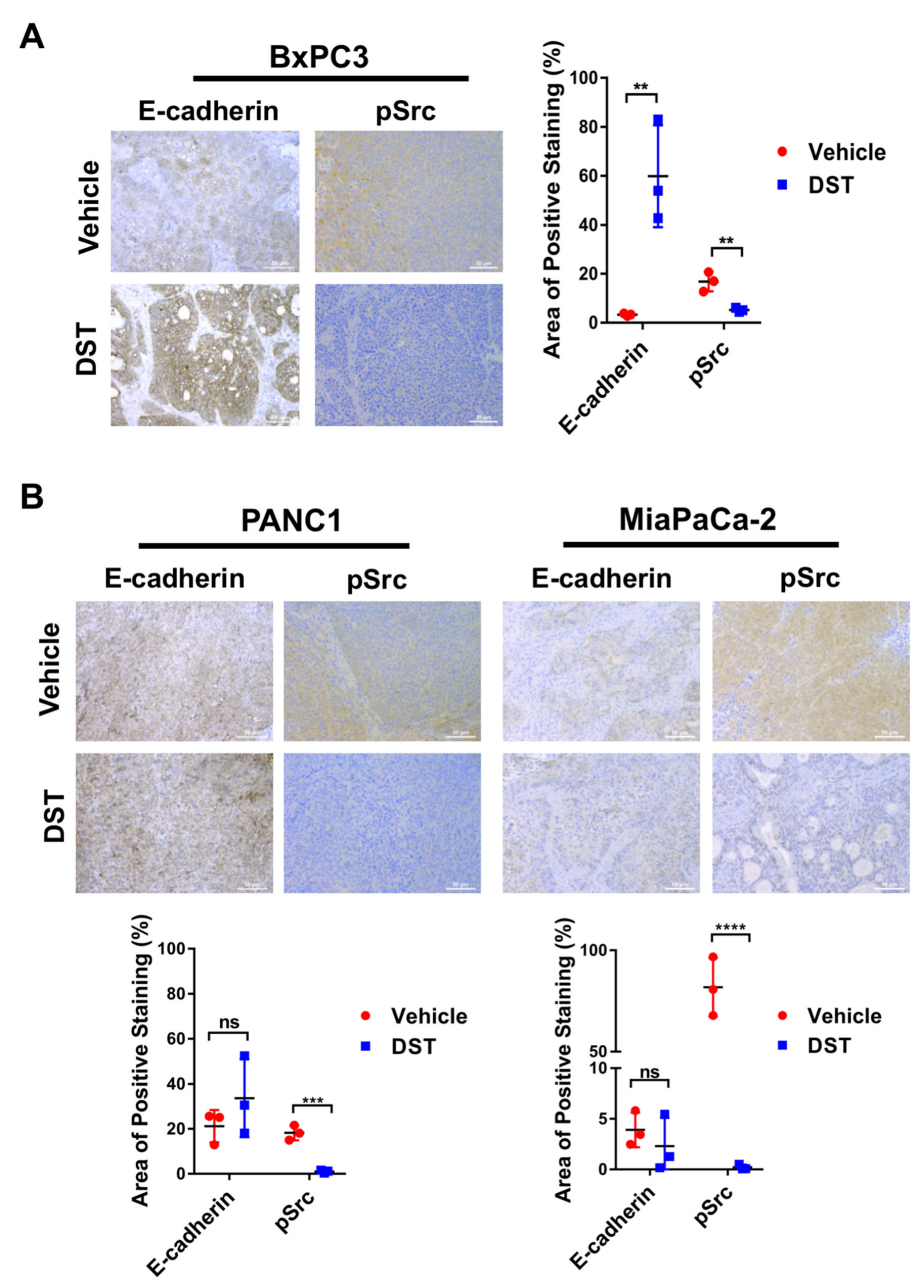
DST treatment restores E-cadherin levels in drug-sensitive BxPC3 xenografts Nude mice were inoculated with BxPC3, PANC1, or MiaPaCa-2 cells (2×10^6^) and treated with vehicle or DST (25 mg/kg) for 14 days before sacrifice. Histological analysis was performed for E-cadherin and pSrc levels in response to treatment in drug-sensitive BxPC3 **(A)** and drug-resistant PANC1 and MiaPaCa-2 **(B)** cell xenografts. Measurements were performed in triplicate and reported as a percentage positive staining of total area. (scale bar = 50μm) ^**^p<0.01, ^***^p<0.001, ^****^p<0.0001, ^ns^ not significant.

To examine if prolonged DST treatment restores E-cadherin expression *in vivo*, a xenograft mouse model was utilized. BxPC3, PANC1, and MiaPaCa-2 cells were inoculated into the flanks of Fox1 *nu-nu* mice. We have previously identified MiaPaCa-2 as a DST-resistant cell line in prior investigations [17]. Flank tumors were grown to 200 – 250 mm^3^ at which point oral gavage with DST at 25 mg/kg or citrate buffer (vehicle) was performed once daily for 14 days. After treatment, mice were sacrificed and tumor levels of E-cadherin were analyzed by immunohistochemistry. In BxPC3, PANC1, and MiaPaCa-2 cell lines, DST treatment successfully reduced pSrc levels compared with control tissue. In drug-sensitive BxPC3 cells, there was limited expression of E-cadherin in control tissue treated with vehicle alone. Consistent with the results of our *in vitro* studies, DST treatment in BxPC3 xenografts significantly increased E-cadherin expression when compared to control tissue (Figure 5A). In drug-resistant PANC1 and MiaPaCa-2 xenografts, there was no difference in E-cadherin expression between control and DST-treated mice, despite a significant decrease in pSrc levels (Figure 5B).

## DISCUSSION

Using both *in vitro* and *in vivo* models, we have demonstrated the therapeutic benefit of Src kinase inhibition in reversing EMT in drug-sensitive PDAC cell lines. Our results indicate that Slug is the main transcription factor affected by DST inhibition in sensitive cell lines, as the dose-dependent decrease in Slug mRNA levels produced by DST treatment was accompanied by a compensatory rise in E-cadherin expression and restoration of an epithelial phenotype, findings which were further validated utilizing Slug-knockdown experiments. In addition to increasing gene transcription and restoring E-cadherin expression, we have demonstrated that DST treatment increases the membranous fraction of both E-cadherin and β-catenin, providing additional insight into how DST treatment curtails EMT in drug-sensitive PDAC cell lines. Using an *in vivo* xenograft model of PDAC, we confirmed our *in vitro* findings. Although pSrc levels were reduced in both cell lines, E-cadherin expression was selectively restored with DST treatment in BxPC3 cells but not in drug-resistant PANC1 or MiaPaCa-2 cells. Furthermore, we have established that there is an inverse relationship between pSrc and E-cadherin expression in human PDAC specimens, indicating the potential translational benefit targeting Src kinase to combat EMT and metastasis in human subjects.

**Figure 6:**
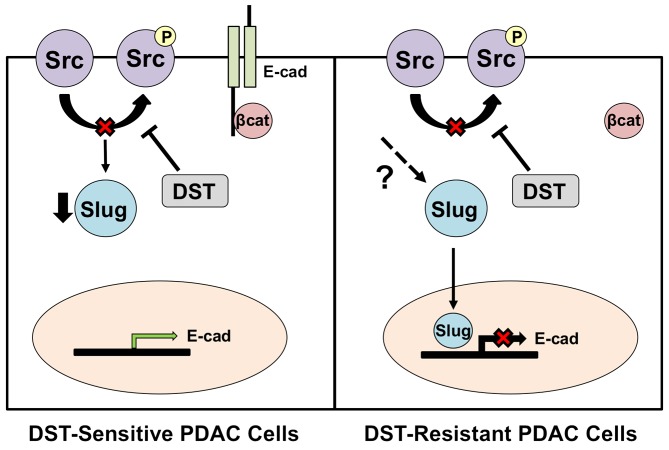
Proposed mechanism of action for DST in drug-sensitive and resistant PDAC cell lines Inhibition of Src kinase in drug-sensitive PDAC cells results in reduced transcription of the pro-EMT transcription factor, Slug. This allows for enhanced E-cadherin transcription and stabilization of the E-cadherin/β-catenin membrane complex. In DST-resistant PDAC cell lines, DST treatment results in a reduction in levels of pSrc, yet Slug transcription is unaffected due to reactivation that is mediated through other unknown mechanisms. This results in continued repression of E-cadherin transcription by Slug in resistant cell lines.

Low tumor E-cadherin expression in surgically resected specimens is associated with advanced TNM stage, early disease metastases, and a poor overall prognosis in PDAC patients [17, 22]. In accordance with our findings in human PDAC specimens, Avizienyte et al demonstrated that elevated Src activity directly decreases E-cadherin levels in colorectal cancer cells through interactions with cellular integrins to destabilize cell-cell adhesion complexes [21]. Trevino et al have previously demonstrated that inhibition of Src kinase by either small interfering RNA (siRNA) or with DST treatment halts the development of PDAC metastases in an orthotopic mouse model [23]. These results were further supported by a study performed by Morton et al which showed DST-treatment suppressed metastatic disease development in genetically-engineered *Pdx1-Cre; Z/EGFP;LSL-Kras^G12D/+^;LSL-Trp53^R172H/+^* mice [24]. Similar data have been reported in colon, liver, and breast cancer cells, where inhibition of the Src kinase pathway reverses EMT, restores E-cadherin expression, and suppresses *in vivo* liver metastasis [25]. However, the mechanism of DST in reducing EMT and restoring E-cadherin levels in PDAC is largely unknown. Our results suggest that Src kinase inhibition reduces the invasive potential of susceptible cells is in part through suppression of Slug-dependent cellular EMT pathways, a finding which has not previously been reported in PDAC and provides a mechanistic rationale for the efficacy of DST in reducing cancer metastases. Slug has previously been identified as a key transcription factor in the process of cancer-associated EMT [26, 27]. Slug is a C2H2-type zinc finger protein which is capable of binding the E-box of the *CDH1* promoter sequence, thus inhibiting E-cadherin transcription [28]. We have previously demonstrated that shRNA-mediated knockdown of Slug induces *CDH1* gene transcription and increases E-cadherin protein levels, findings which were further validated in the present study [29]. Additionally, a recent investigation by Li et al confirmed that Slug expression represses E-cadherin in pancreatic cancer [30]. In a study by Conacci-Sorrell et al, regulation of Slug activity was predominately governed by β-catenin translocation in colon cancer cells. Disruption of E-cadherin-mediated cell-cell contact resulted in β-catenin migration to the nucleus and a subsequent increase in Slug activity and downregulation of E-cadherin [31]. Vultur et al showed additionally that Src kinase inhibition prevents nuclear β-catenin translocation through stabilization of membrane cadherins, leading to enhanced E-cadherin expression in human breast cancer cells [32]. These data support our findings in this study, which show that DST leads to an increase in membranous fraction of β-catenin which correlated with a decrease in Slug activation and restoration of E-cadherin expression in drug-sensitive cells. This suggests that Src kinase inhibition may reverse EMT in part through stabilization of the E-cadherin/β-catenin membrane complex, thus preventing β-catenin translocation and Slug activation in drug-sensitive PDAC cells (Figure 6).

This investigation clearly demonstrates that not all PDAC cell lines are susceptible to the anti-tumor effects of DST. BxPC3 cells, which do not contain activating mutations in the *KRAS* proto-oncogene, were highly susceptible to Src kinase inhibition, whereas *KRAS^G12D^*–mutant PANC1 and MiaPaCa-2 cells were resistant to therapy [33]. However, SW1990, which is *KRAS-*mutant, was sensitive to DST-mediated suppression of Slug and restoration of E-cadherin in our study, suggesting that aberrant activation of the Ras/Raf pathway alone does not confer resistance to DST. This is consistent with previous reports which demonstrated that common genetic mutations in PDAC, including activating Ras mutations, p53 mutations, or Smad4/DPC4 mutations, do not predict response to DST treatment [17, 34]. Differences in susceptibility may be explained by examining the diverse transcription factors regulating EMT in PDAC. The lack of susceptibility of PANC1 cells to DST therapy suggests that EMT pathway activation in these cells may be mediated in a Slug-independent manner [17, 34]. Multiple other transcription factors aside from Slug have been shown to regulate EMT in PDAC, including Snail, Zeb1/2, and TWIST [35]. Of these, recent evidence suggests that the Zeb1 transcription factor is the master regulator of EMT in PDAC and its activation can drive expression of other pro-EMT genes. In a study by Krebs et al, genetic deletion of Zeb1 in *Pdx1a^cre^;LSL-Kras^G12D/+^;LSL-Tp53^R172H/+^* transgenic mice restored an epithelial phenotype, decreased invasion, and inhibited the development of metastatic disease *in vivo*. Furthermore, Zeb1 knockout resulted in dramatically reduced mRNA levels of Snail, Slug, Zeb2, and TWIST, suggesting that elevated Zeb1 activity can independently drive expression of these transcription factors [36]. Zeb1 is expressed in PANC1 drug-resistant cells, but not in sensitive BxPC3 cells (Supplementary Figure 2B). In our present study, we show transcription of Zeb1 in PANC1 cells is not reduced by DST treatment (Supplementary Figure 2A). This suggests that one potential resistance mechanism to Src kinase inhibition may be mediated through constitutive activation of Zeb1, thus leading to continued Slug expression despite DST therapy. However, the present study does not specifically address the role of Zeb1 in overcoming Src inhibition and elucidation of the precise biologic mechanisms which confer resistance to DST warrant further investigation.

The variable response to Src kinase inhibition observed in our study is mirrored in clinical trials utilizing DST for the treatment of PDAC. In a recent double-blind phase 2 study, the addition of DST to gemcitabine chemotherapy failed to show an improvement in overall survival or progression free survival in patients with locally advanced, non-metastatic PDAC [37]. However, DST treatment did achieve stable disease in two of eight patients in a phase 1 study, further suggesting that the genetic heterogeneity of PDAC tumors heavily influences therapeutic response to DST [20]. Therefore, identifying the cellular biomarkers which accurately predict response to DST will be critical in determining the subset of patients who may respond to Src kinase inhibition. Our results suggest that a restoration in E-cadherin expression with DST treatment may be relevant biomarker for sensitivity to Src kinase inhibition in PDAC patients. E-cadherin has previously been identified as a potential biomarker for treatment response to EGFR inhibition in non-small cell lung cancer [38]. Furthermore, elevated circulating levels of E-cadherin fragmentation products have been reported to correlate with disease progression in patients with prostate cancer, providing a platform for non-invasive monitoring of tumor E-cadherin levels during treatment [39]. Abnormal E-cadherin expression occurs in over 50% of PDAC patients and therefore may be a promising biomarker to assess which subset of tumors are susceptible to Src kinase inhibition in clinical studies and should be investigated in further studies [40].

In conclusion, we have demonstrated that DST treatment reverses Src-mediated E-cadherin repression and results in a concomitant decrease in the expression of the pro-EMT transcription factor Slug in drug-sensitive PDAC cells. These data are the first to show the potential therapeutic benefit of Src kinase inhibition in reversing EMT through Slug inhibition to improve patient outcomes in PDAC. As clinical studies utilizing DST are currently ongoing, our data show that PDAC tumor response to DST is variable and identification of biomarkers to predict response to Src kinase inhibition are essential to allow clinicians to personalize treatment regimens based on specific tumor biology. These results suggest E-cadherin may be utilized as a potential biomarker for response to DST therapy and should be explored in future studies.

## MATERIALS AND METHODS

### Cell lines and reagents

Human pancreatic cancer cell lines Capan-2, PANC1, MiaPaCa-2, SW1990 and BxPC3 were obtained from the American Type Culture Collection (ATCC). Tumor cells were maintained according to ATCC guidelines. Mouse monoclonal antibodies against E-cadherin and β-catenin were purchased from BD Biosciences Pharmingen. Dasatinib (BMS-354825) was kindly provided by Richard Smykla from Bristol-Myers-Squibb Oncology (Princeton, NJ, USA).

Cell authentication was performed by using STR DNA profiling (latest date: June 16, 2016) and cell lines tested negative for Mycoplasma via Genetica cell line testing (Burlington, NC, USA) using eMYCO plus kit (iNtRON Biotechnology). Cells with relative low passage numbers (< 20) were used in the study. ATCC cell lines were characterized and were free of Mycoplasma contamination, tested by Hoechst DNA stain (indirect) and agar culture (direct) methods.

### Western blot analysis

Western blot analysis was performed using standard methods previously described [41]. In brief, after treatment, cells were washed and scraped off the culture dishes. Cell pellets were collected by centrifuge and lysed in RIPA buffer (0.1% SDS, 50 mM Tris·HCl, 150 mM NaCl, 1% NP-40, and 0.5% Na deoxycholate) with protease inhibitor cocktail (Sigma, St. Louis, MO, USA). Cell lysates were sonicated and centrifuged to collect supernatant. The collected supernatant was quantified for protein concentration. The same quantities of protein from all the samples were loaded onto a SDS-PAGE gel. After running the gel, the proteins were transferred to a PVDF membrane and probed for the proteins of interest.

### Immunofluorescence staining

Cells were fixed and stained according to published methods [17]. Treated cells were fixed and incubated with the following antibodies: anti-E-cadherin (R&D Systems, 1:500), anti-β-catenin (1:500, R&D Systems), anti-Zeb1 (1:200, Abcam), and F-actin (Cell Signaling). Following primary incubation, conjugation with FITC (Sigma Biochemicals), Cy3 (Sigma Biochemicals), or Alexa Fluor 594 (Invitrogen) secondary antibodies was then performed. The cells were imaged with a Zeiss Axiophot microscope. Images were merged and analyzed using NIH Image J software.

### Cell invasion assay

Invasion analysis was performed using standard methods previously described [42]. In brief, the upper chamber of 8 μm pore transwells were overlaid with 40 μL (~100 μg) of diluted Matrigel (BD Biosciences). 3×10^4^ cells were suspended in serum free media containing either DMSO or DST (100 nmol/L) and seeded into the upper chamber of transwell insert. Medium containing 10% fetal bovine serum was used as a chemoattractant in the lower chamber. Cells were allowed to invade for 24 hours prior to fixation with 4% paraformaldehyde and staining with 1% crystal violet. Cell density was counted using NIH Image J software.

### RNA isolation and reverse transcription (RT)-PCR analyses

Cells were treated with DMSO or dasatinib (1-1000 nmol/L) for 12 hours, and total RNA was isolated with TRIzoI™ reagent (Invitrogen). The RT reactions were conducted at 42-44°C for 1 hour and contained 1 μg of total RNA and 2.5 μL cDNA mixtures. The PCR conditions were: 2.5 min at 95°C; followed by 27 cycles of 95°C for 30 s; 55°C for 1 min, and 72°C for 1 min with a final extension at 70°C for 5 min. The following primers were used: E-cadherin, 5′-CCCACCACGTACAAGGGTC-3′ (sense), 5′-ATGCC ATCGTTGTTCACTGGA-3′ (antisense); Slug, 5′-ATA CCACAACCAGAGATCCTCA-3′ (sense) and 5′-GACT CACTCGCCCCAAAGATG-3′ (antisense); LEF-1, 5′-AA TGAGAGCGAATGTCGTTGC-3′ (sense) and 5′-GCTG TCTTTCTTTCCGTGCTA-3′ (antisense); Snail, 5′-AATC GGAAGCCTAACTACAGCG-3′ (sense) and 5′-GTCCC AGATGAGCATTGGCA-3′ (antisense); TWIST, 5′-GTC CGCAGTCTTACGAGGAG-3′ (sense) and 5′-GCT TGAGGGTCTGAATCTTGCT-3′ (antisense); ZEB1, 5′-TCCATGCTTAAGAGCGCTAGCT-3′ (sense), 5′-ACC GTAGTTGAGTAGGTGTATGCCA-3′ (antisense); GAPDH, 5′-ACCACAGTCCATGCCATCAC-3′ (sense), and 5′-TCCACCACCCTGTTGCTGTA-3′ (antisense). Relative gene expression was calculated by normalizing each treatment value to corresponding GAPDH signal intensity, then reported relative to control signal intensity.

### Transient transfection and promoter study

Transfection was as described previously [43]. Cells were co-transfected with a firefly E-cadherin luciferase promoter-reporter construct (pGL2 E-cadh3/Luc containing E-cadh 5’ flanking sequences of 1485 bp) and E-box mutant E-cadherin (Addgene plasmid 19291) luciferase construct (kindly provided by Dr. E. R. Fearon) [27, 44] with an equal amount of total DNA by using Lipofectamine Plus reagents (Invitrogen). Luciferase activity was normalized to β-galactosidase activity and the relative luciferase activity was presented.

### Slug gene-knockdown in PDAC cells

Generation of Slug knockdown was performed as described previously [29]. In brief, lentiviral shRNA vector pGIPZ with either targeting sequences for human Slug with Open Biosystems pGIPZ-based short hairpin RNA (shRNA) (Clone IDs: 153125 and 153128) or non-silencing control sequences were used. Cells were transfected with FUGENE 6 transfection reagent (Roche) following the manufacturer's instruction. Lentiviral particles were prepared by co-transfecting three plasmids into 293T cells, including pMD2.G, psPAX2 and lentivectors. BxPC3 cells were transduced with lentiviral vectors at an MOI (multiplicity of infection) of 20 supplemented with polybrene (6 μg/ml) for 18-20 hours, and GFP expression was confirmed by FACS Caliber flowcytometer (BD Biosciences). The cells were then selected for 7 days with puromycin (2.5 μg/ml). The colonies obtained from single cells were screened for the expression of Slug by qRT-PCR.

### Immunohistochemistry

Tissues were fixed and immunostained using antibodies against E-cadherin and pSrc (T416, Cell Signaling). Tissue slides were deparaffinized, antigen retrieval was carried out in citrate buffer (pH = 6.0) under pressure for 15 minutes, and endogenous peroxidase activity was blocked by incubating with 3% H_2_O_2_ for ten minutes. The sections were stained with primary antibodies at described concentrations and developed using DAB substrate (Vector, Burlingame, CA, USA). Immunostained slides were imaged using Leica microscope (Leica Microsystems, Inc. Buffalo Grove, IL, USA) and quantified using Image J. Values were calculated and reported as area positive (%) protein staining.

### *In vivo* xenograft mouse model of PDAC

Athymic nude mice—Foxn1-*nu/nu* (4–5 weeks old)—were purchased from Harlan Sprague Dawley, Inc. Subcutaneous tumors were established by injecting 2×10^6^ BxPC3, PANC1, or MiaPaCa-2 cells into the flank of a 6-week-old Fox1-*nu/nu* mouse (n=5 in each group) as previously detailed [45]. Treatment was initiated when the subcutaneous (s.c.) tumors reached 200 – 250 mm^3^ size. DST (25 mg/kg/day) or vehicle (citrate buffer) was administered by oral gavage. The subcutaneous tumor volume and percent body weight change was recorded as previously described [45, 46]. Growth curves for tumors were plotted as the mean volume ± standard deviation (s.d.) of tumors for mice from each group. At the end of the study, animals were sacrificed and their primary tumors were removed.

All experiments were performed in compliance with the regulations and ethical guidelines for experimental and animal studies of the Institutional Animal Care and Use Committees at the University of Miami (Miami, FL, USA) (Protocol #15-057, 15-099 and 18-081).

### Statistical analysis

Descriptive statistics were calculated using Microsoft Excel and Prism software (Graphpad Software Inc.). Results are shown as values of mean ± s.d. unless otherwise indicated. Statistical analyses of immunohistochemistry data were performed using Student's *t*-test with P < 0.05 taken as significant, except where indicated otherwise. One-way ANOVA was used to assess the differences between experimental groups unless otherwise indicated. Quantification of IHC images, RT-PCR, and Western blot analyses was performed using ImageJ software (NIH).

## SUPPLEMENTARY MATERIALS AND FIGURES


