# Supplementary material for: Src kinase inhibition restores E-cadherin expression in dasatinib-sensitive pancreatic cancer cells
Source: Oncotarget. 2019 Feb 1;10(10):1056–69. doi: 10.18632/oncotarget.26621 (PMC6383685; doi:10.18632/oncotarget.26621)
Supplement: Supplementary file 1 [file oncotarget-10-1056-s001.pdf]

Src kinase inhibition restores E-cadherin expression in dasatinib-sensitive pancreatic cancer cells

SUPPLEMENTARY MATERIALS

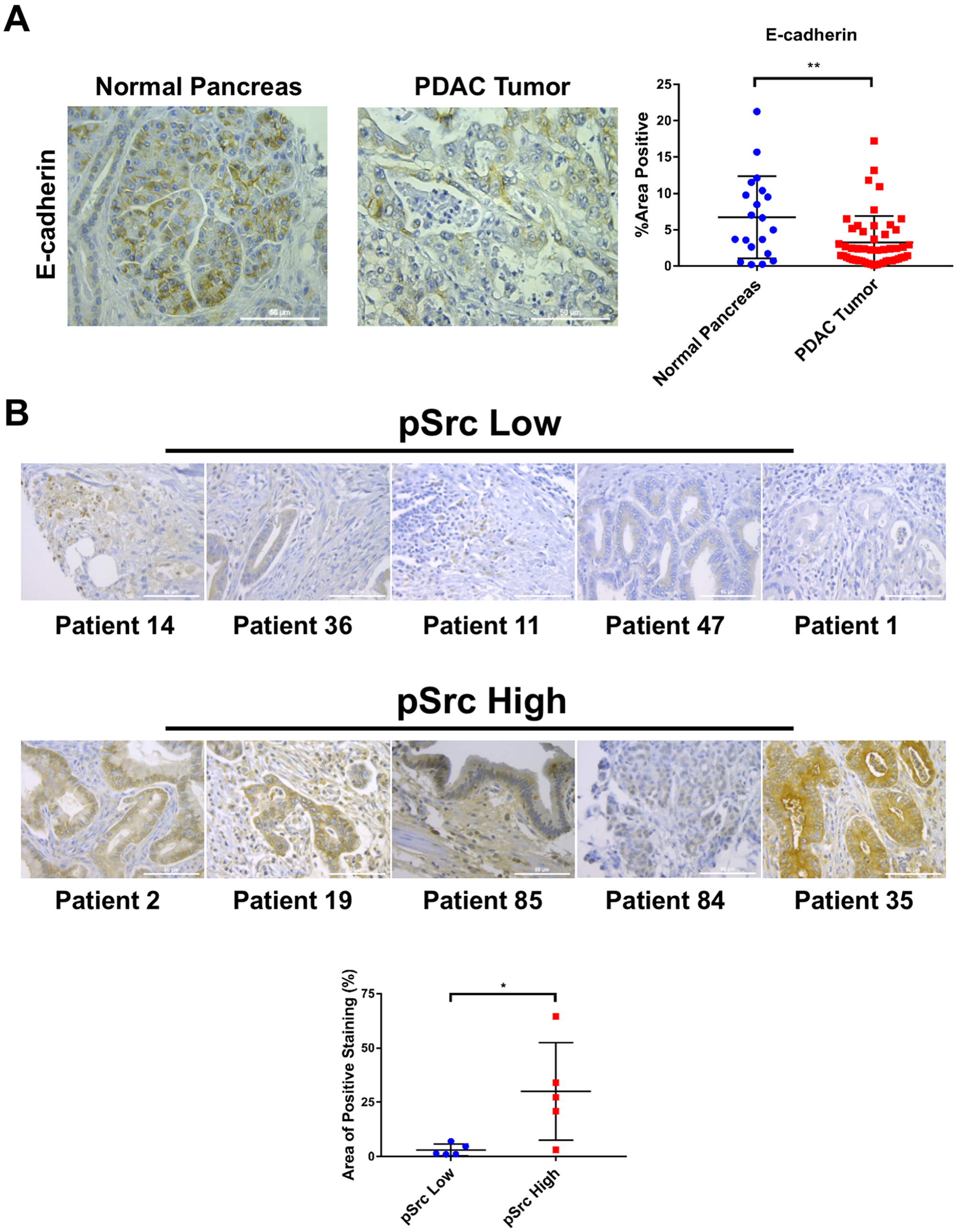

Supplementary Figure 1: Tissue microarray (TMA) of normal pancreas (N=20) and human PDAC tumor (N=50) samples were analyzed for E-cadherin expression **(A)**. Patient tissue specimens were stained for pSrc expression (N=10) and stratified into $pSrc^{low}$ and $pSrc^{high}$ groups for further analysis (scale bar = 50µm) **(B)**. Total pSrc expression between was reported as a percentage area of positive pSrc staining (lower panel). $^{*}p<0.05$, $^{**}p<0.01$.

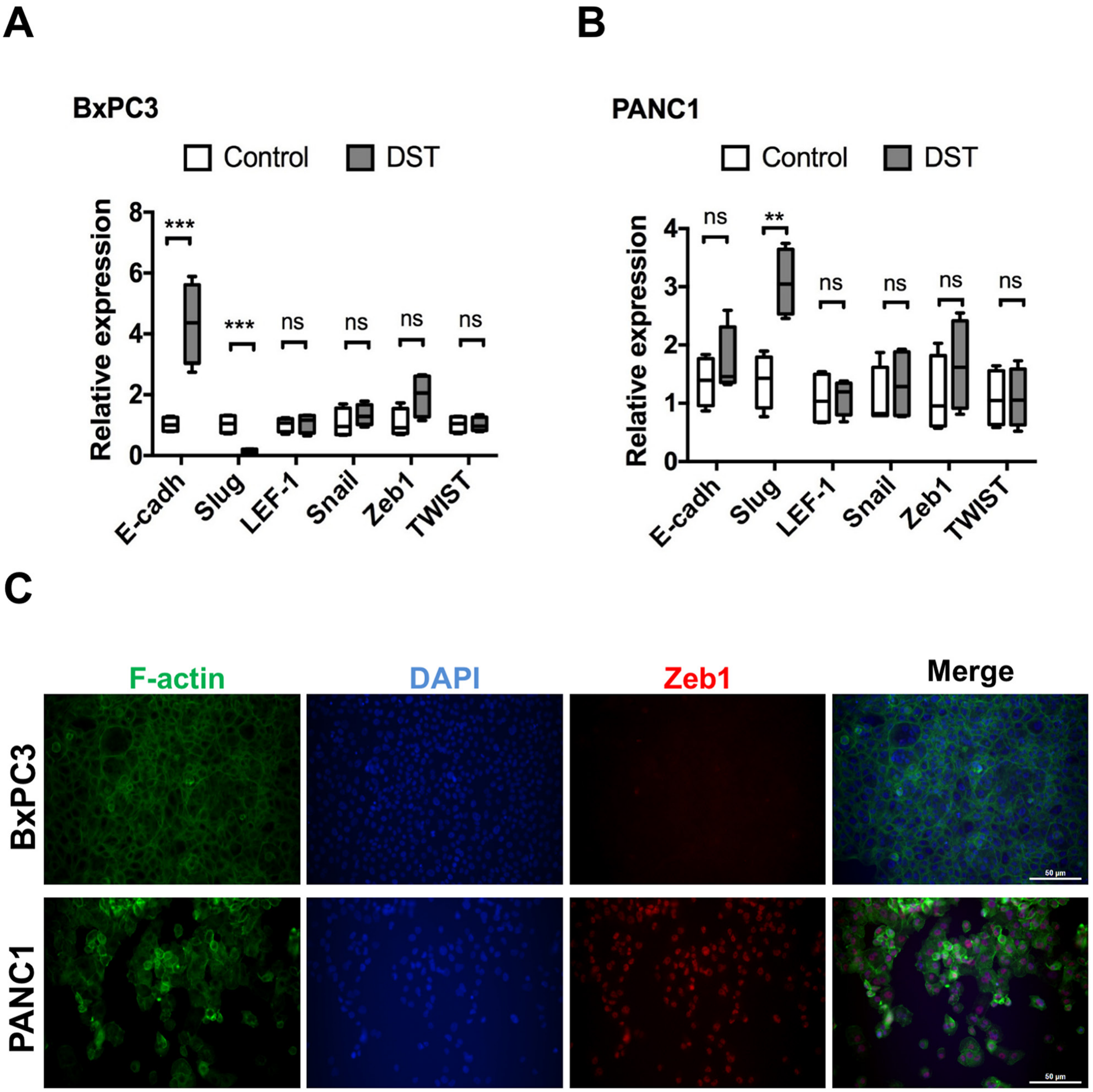

Supplementary Figure 2: BxPC3 **(A)** and PANC1 **(B)** cells were treated with DMSO or DST (100 nmol/L) for 24 h and transcription levels of E-cadherin, Slug, LEF-1, Snail, Zeb1, and TWIST were measured by qPCR. Levels of Zeb1 (red) were determined in BxPC3 and PANC1 cell lines by immunofluorescence **(C)**. Cells were counterstained with DAPI (blue) and F-actin with Phalloidin (green) (scale bar = 50µm).
